# Oculogyric Crisis in Early Pregnancy: Lessons Learned From a Rare Adverse Effect of Metoclopramide

**DOI:** 10.7759/cureus.78522

**Published:** 2025-02-04

**Authors:** Kajananan Sivagurunathan, Prashanthan Kaneshamoorthy, Nalayini Jegathesan, Peranantharajah Thampipillai

**Affiliations:** 1 Internal Medicine, Teaching Hospital Jaffna, Jaffna, LKA

**Keywords:** acute dystonia, extrapyramidal side effects, metoclopramide, oculogyric crisis, pregnancy, trihexyphenidyl

## Abstract

Metoclopramide is a widely prescribed antiemetic, particularly in pregnancy, for managing nausea and vomiting. While it is considered safe in most cases, rare extrapyramidal side effects such as acute dystonia with oculogyric crisis can occur. Here, we present a case of a 12-week pregnant woman who developed acute dystonia with oculogyric crisis after treatment with metoclopramide. Oral trihexyphenidyl (Benzhexol) led to rapid symptom resolution. This case emphasizes the need for awareness of rare adverse drug reactions and the importance of early recognition to prevent further complications.

## Introduction

Metoclopramide is frequently used to treat nausea and vomiting in pregnancy due to its effectiveness and general safety profile. Metoclopramide inhibits dopamine receptors (D2 receptors) in the brain, particularly in the chemoreceptor trigger zone (CTZ), which is responsible for inducing nausea and vomiting. It also has a prokinetic effect by inhibiting dopamine receptors in the gastrointestinal tract, which accelerates gastric emptying [1]. Metoclopramide can cross the blood-brain barrier, which is why it can cause central side effects, such as extrapyramidal symptoms (e.g., tardive dyskinesia, dystonia including oculogyric crisis, and akathisia) and sedation [2].

Acute dystonia is a movement disorder characterized by the sudden onset of sustained muscle contractions, leading to twisting, repetitive movements, or abnormal postures. It is a type of extrapyramidal symptom, often caused by using dopamine antagonists, including antipsychotic medications and antiemetics like metoclopramide. Here, we present a case of metoclopramide-induced acute dystonia with oculogyric crisis in a pregnant woman at 12 weeks of gestation.

## Case presentation

A 24-year-old primigravida, at 12 weeks of gestation, presented to the outpatient department with nausea and vomiting for three days. She had experienced pregnancy-related nausea without vomiting during the early gestational period (around eight to 10 weeks) but was able to manage it herself and did not seek medical advice. This time, at 12 weeks of gestation, she had been vomiting one to three times daily for the past three days. She experienced nausea lasting for less than an hour, occurring three to four times per day. She did not report headaches, aura, or visual symptoms. There was no history of dehydration, weight loss, or other features suggestive of hyperemesis gravidarum. She was prescribed oral metoclopramide 10 mg three times daily, along with oral famotidine 20 mg twice daily and oral thiamine 100 mg daily. Empirical thiamine supplementation was included to prevent deficiency due to ongoing vomiting for three days, even with mild vomiting. She was assessed to be tolerating oral intake despite mild ongoing vomiting.

**Figure 1:**
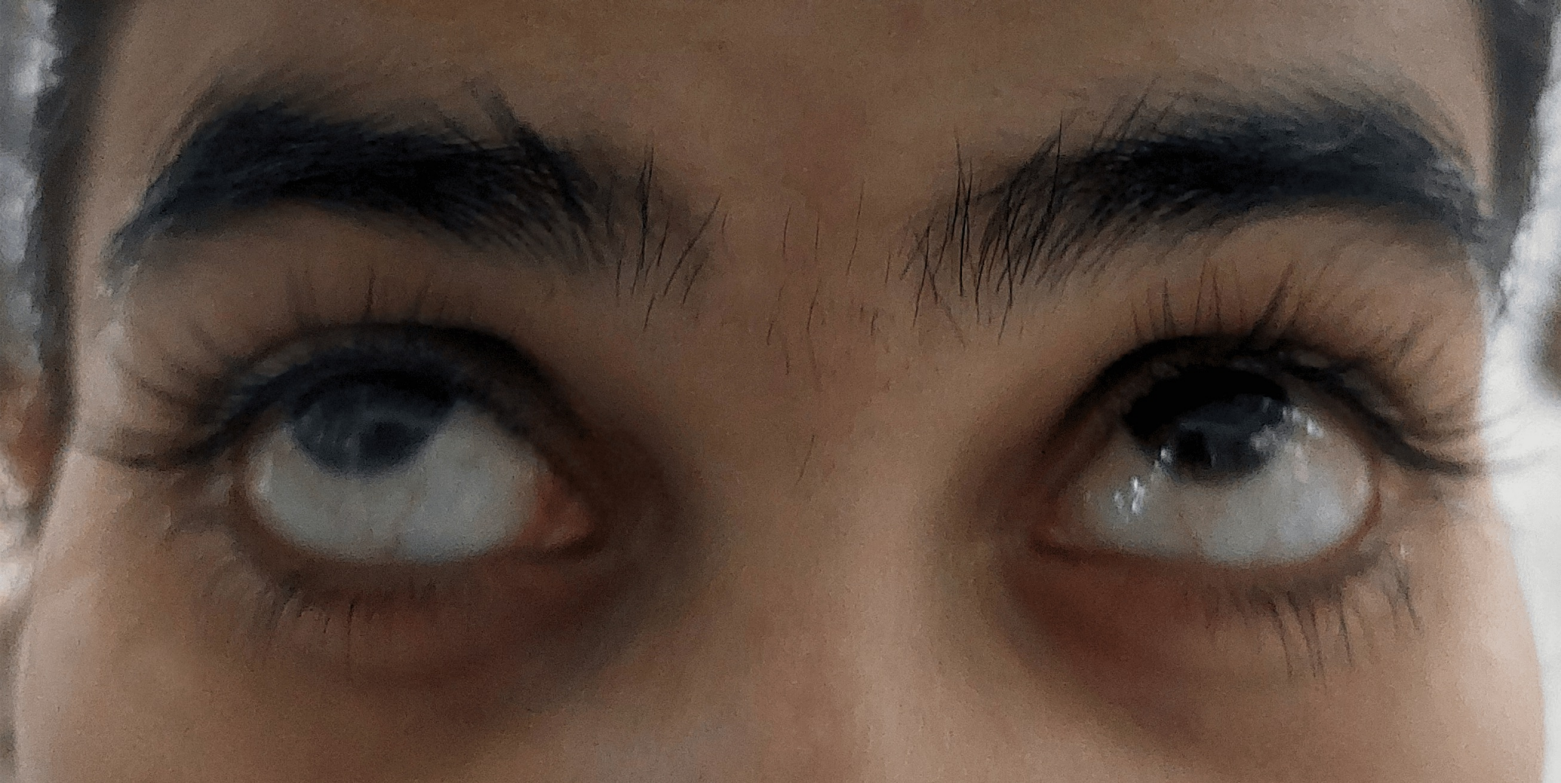
Oculogyric crisis, characterized by a sustained upward deviation of the eyes, is described in this case of a pregnant woman receiving metoclopramide.

After two days of taking metoclopramide, she presented to the casualty medical ward with complaints of involuntary upward rolling of the eyes (Figure 1) and painful neck extension, which began three hours before the presentation. She described an inability to move her eyes back to a neutral position, and her neck extension was associated with significant discomfort and pain. She did not have a personal or family history of any movement disorder. She had no comorbidities or history of allergies. On examination, she was alert with a normal Glasgow Coma Scale (GCS) score, and there were no other neurological abnormalities. Her neck muscles were tense due to dystonic spasms. Her other vital parameters including blood pressure, pulse rate, respiratory rate, and oxygen saturation were normal.

Blood investigations, including full blood count, inflammatory markers, serum creatinine, liver enzymes, and serum electrolytes, were within normal limits. Based on her clinical presentation, a diagnosis of metoclopramide-induced acute dystonia with oculogyric crisis was made. We conducted a causality assessment using the Naranjo Scale to evaluate the likelihood that metoclopramide caused the oculogyric crisis in this patient. The Naranjo Scale score was 7 as shown in Table 1, indicating a probable association between the drug and the adverse event. The offending drug was discontinued. Due to the unavailability of parenteral benztropine, oral trihexyphenidyl (Benzhexol) 2 mg was administered despite ongoing nausea. Given the absence of active vomiting at the time of treatment and the high oral bioavailability of trihexyphenidyl, this was considered a reasonable alternative. Her symptoms began to resolve within two to three hours of treatment. She reported complete relief from her symptoms by the time of discharge the following day. She was advised to avoid metoclopramide in the future.

**Table 1:** Naranjo adverse drug reaction probability scale Total score: ≥9 = a definitive adverse drug reaction, 5-8 = a probable adverse drug reaction, 1-4 = a possible adverse drug reaction, ≤0 = a doubtful adverse drug reaction

Question	Yes	No	Do not know	Score
1. Are there previous conclusive reports on this reaction?	+1	0	0	+1
2. Did the adverse event appear after the suspected drug was administered?	+2	-1	0	+2
3. Did the adverse reaction improve when the drug was discontinued, or a specific antagonist was administered?	+1	0	0	+1
4. Did the adverse event reappear when the drug was re‐administered?	+2	-1	0	0
5. Are there alternative causes (other than the drug) that could on their own have caused the reaction?	-1	+2	0	+2
6. Did the reaction reappear when a placebo was given?	-1	+1	0	0
7. Was the drug detected in blood (or other fluids) in concentrations known to be toxic?	+1	0	0	0
8. Was the reaction more severe when the dose was increased or less severe when the dose was decreased?	+1	0	0	0
9. Did the patient have a similar reaction to the same or similar drugs in any previous exposure?	+1	0	0	0
10. Was the adverse event confirmed by any objective evidence?	+1	0	0	+1
Total Score				+7

## Discussion

Metoclopramide, a dopamine receptor antagonist, is one of the most common drugs used to treat nausea and vomiting. Metoclopramide is associated with a range of adverse effects, with extrapyramidal side effects, including acute dystonia and oculogyric crisis, being among the more common as in this case [3]. These adverse effects result from an imbalance between dopaminergic and cholinergic neurotransmission in the nigrostriatal pathway. The imbalance is due to inhibition of dopamine D2 receptors by metoclopramide leading to unopposed cholinergic activity. As per the SPC, the incidence of extrapyramidal side effects with metoclopramide is reported to be between ≥1/100 and <1/10 (1-10%) [3]. Notably, acute dystonic reactions do not appear to correlate with the drug’s dosage, indicating idiosyncratic sensitivity in certain individuals [4]. These reactions are more frequent in female patients, children and adults less than 30 years of age [5]. Symptoms following the use of metoclopramide may take as long as 36 hours to manifest [5]. In many developing countries like Sri Lanka, studies on adverse reactions caused by metoclopramide remain limited. The true frequency of these reactions is likely underestimated due to underreporting [5].

Several case reports describe metoclopramide-induced dystonia with oculogyric crises. A case report described a 13-year-old female who presented with involuntary upward and lateral deviation of the eyes after the use of metoclopramide for two days [6]. She has an oculogyric crisis with neck dystonia as our patient. She improved after 30 minutes of starting intravenous diphenhydramine. Another case report by Arumugam J et al. described a 14-year-old girl who presented with a diagnostic dilemma of encephalitis [7]. She took metronidazole, metoclopramide, paracetamol, and oral rehydration solutions (ORS) for fever, loose stools, and vomiting. She developed agitation, headache, dysphagia, upward eye deviation, and neck hyperextension, mimicking meningeal irritation signs. Investigations including computed tomography of the brain were normal. The metoclopramide-induced dystonic reaction was suspected, and she was treated with oral diphenhydramine. Symptoms resolved within 12 hours.

CYP2D6 is an enzyme involved in serotonin synthesis. It plays a role in inhibiting dopamine tone. Individuals who are poor metabolizers of CYP2D6 may have a higher risk of dystonic reactions to metoclopramide [8-9]. A study reported two pregnant women who were identified as CYP2D6 poor metabolizers. They developed metoclopramide-induced acute dystonic reactions [8]. Elevated oestrogen levels during pregnancy are known to modulate dopamine receptor sensitivity, potentially increasing the likelihood of such adverse reactions [8]. This mechanism may explain the reaction observed in our case. Thus, hormonal changes during pregnancy may increase the risk of dystonic reactions to dopamine antagonists like metoclopramide.

The management of acute dystonia involves discontinuing the offending drug and administering anticholinergic medications. Benztropine and diphenhydramine are the most commonly used drugs, as they effectively restore the dopaminergic-cholinergic balance in the basal ganglia [10]. In our case, oral trihexyphenidyl (Benzhexol) led to rapid symptom improvement, consistent with findings in the literature. Regarding safety in pregnancy, benztropine (category B2) has limited human data but no proven fetal harm in animal studies [11]. Trihexyphenidyl (category C) may pose risks based on animal studies and should be used only if the benefits outweigh the risks [12]. Diphenhydramine (category B) is generally considered safe [13]. No specific drug of choice exists in pregnancy, and selection should be based on availability in the hospital and a risk-benefit assessment.

First-line antiemetics for vomiting in pregnancy include H1-antihistamines, phenothiazines, and doxylamine/pyridoxine (Xonvea®) [14]. Second-line agents include ondansetron and metoclopramide. However, the use of phenothiazines and metoclopramide has been associated with extrapyramidal symptoms, including oculogyric crises [14]. In patients at higher risk of developing extrapyramidal symptoms, alternative options should be considered, excluding these two agents. However, there are no clear guidelines on the optimal choice of antiemetics in such cases, highlighting the need for further research to guide clinical decision-making.

This case emphasizes the importance of awareness among healthcare providers regarding the rare but significant side effects of metoclopramide. Early recognition of drug-induced dystonic reactions is crucial to prevent unnecessary distress and avoid extensive investigations. Furthermore, there is a need for larger studies in pregnancy to better understand the risk factors, incidence, and management of these adverse effects, as prescribing metoclopramide in pregnancy is more common.

## Conclusions

This report adds to the growing body of literature on metoclopramide-induced acute dystonia with oculogyric crisis, particularly in pregnant women. It emphasizes the need for clinical awareness, early diagnosis, and prompt treatment with anticholinergic therapy to ensure favourable outcomes. Alternative antiemetics should be considered in high-risk populations, and further research is warranted to establish comprehensive guidelines for the safe use of metoclopramide during pregnancy.
